# Effects of Sample Selection Bias on the Accuracy of Population Structure and Ancestry Inference

**DOI:** 10.1534/g3.113.007633

**Published:** 2014-03-17

**Authors:** Suyash Shringarpure, Eric P. Xing

**Affiliations:** * Department of Genetics, Stanford University, Stanford, California 94305; † School of Computer Science, Carnegie Mellon University, Pittsburgh, Pennsylvania 15213

**Keywords:** admixture analysis, global ancestry, biased sampling, population structure

## Abstract

Population stratification is an important task in genetic analyses. It provides information about the ancestry of individuals and can be an important confounder in genome-wide association studies. Public genotyping projects have made a large number of datasets available for study. However, practical constraints dictate that of a geographical/ethnic population, only a small number of individuals are genotyped. The resulting data are a sample from the entire population. If the distribution of sample sizes is not representative of the populations being sampled, the accuracy of population stratification analyses of the data could be affected. We attempt to understand the effect of biased sampling on the accuracy of population structure analysis and individual ancestry recovery. We examined two commonly used methods for analyses of such datasets, ADMIXTURE and EIGENSOFT, and found that the accuracy of recovery of population structure is affected to a large extent by the sample used for analysis and how representative it is of the underlying populations. Using simulated data and real genotype data from cattle, we show that sample selection bias can affect the results of population structure analyses. We develop a mathematical framework for sample selection bias in models for population structure and also proposed a correction for sample selection bias using auxiliary information about the sample. We demonstrate that such a correction is effective in practice using simulated and real data.

Population stratification is an important task in genetic analyses. It provides information about the genetic ancestry of individuals and evolutionary history of populations (Rosenberg *et al.* 2002) and can be used to correct for confounding effects in genetic association studies (Price *et al.* 2006). A large number of human genetic datasets such as the HAPMAP (Gibbs 2003), Human Genome Diversity Project (Cavalli-Sforza 2005) along with a smaller number from other organisms are available for study. Datasets that sample a number of individuals from a specific region also have been analyzed to look for evidence of population stratification. These datasets contain individuals from geographically and ethnically diverse populations. Due to practical constraints, only a small number of individuals from each population are genotyped, and the resulting data are a sample from the entire population. This often means that the sample selected for analysis is a biased sample from the underlying populations. This problem is also encountered when multiple datasets are combined to detect population structure analysis with better resolution.

We hypothesize that if the distribution of sample sizes is not representative of the populations being sampled, the accuracy of population stratification analyses of the data could be affected because a fundamental assumption of statistical learning algorithms is that the sample available for analysis is representative of the entire population distribution. Although most algorithms are robust to minor violations of this assumption, sampling bias in the case of genetic datasets may be too large for algorithms to accurately recover stratification.

In this work, we develop a mathematical framework for modeling sample selection bias in genotype data. Our experiments on simulated data show that accuracy of population stratification and recovery of individual ancestry are affected to a large extent by the sampling bias in the data collection process. Both likelihood-based methods and eigenanalysis show sensitivity to the effects of sampling bias. We show that sample selection bias can affect population structure analysis of genotype data from cattle. We also propose a mathematical framework to correct for sample selection bias in ancestry inference reduce its effects on ancestry estimates. We show how such a correction can be implemented in practice and demonstrate its effectiveness on simulated and real data.

## Related work

We briefly examine methods that can be used for population structure analysis and the factors that affect their accuracy. We also examine related work on addressing the problem of sample selection bias in different contexts.

### Methods of population structure analysis

A variety of methods have been developed for detecting population structure. The two main classes of methods used for detecting population structure are model-based methods and eigenanalysis. Model-based methods use an explicit admixture model of how the population sample was formed from its ancestral populations. The STRUCTURE model by Pritchard *et al.* (2000) was one of the early methods of this class that is commonly used. Extensions to the STRUCTURE method have been proposed to account for other observed evolutionary processes (Falush *et al.* 2003; Huelsenbeck and Andolfatto 2007; Shringarpure and Xing 2009). The frappe method by Tang *et al.* (2005) and the ADMIXTURE method by Alexander *et al.* (2009) are alternative ways of solving the optimization problem underlying the STRUCTURE model. They allow us to efficiently analyze datasets of large size.

The eigenanalysis methods proposed by Price *et al.* (2006) and Patterson *et al.* (2006) project genetic data from individuals into a low-dimensional space formed from the eigenvectors of the genetic sample. Since they do not assume a specific model of population evolution, they can be used in a variety of evolutionary scenarios.

Engelhardt and Stephens (2010) showed that the model-based approach and the eigenanalysis-based approach to stratification could both be interpreted as different ways of factorizing the genotype matrix of the given data, which suggests that both methods are related despite their apparent differences.

### Factors affecting the accuracy of stratification

A number of factors are known to affect the accuracy of population stratification and individual ancestry recovery. In one of the early works on model-based methods for population stratification, Pritchard *et al.* (2000) showed that the number of loci available for analysis had a significant effect on the recovery of individual ancestry using STRUCTURE. Kaeuffer *et al.* (2007) studied the effect of linkage disequilibrium on recovery of population structure using simulated data. McVean (2009) suggested an interpretation of the eigenanalysis method that is the basis of the EIGENSOFT method in terms of the coalescence times of individuals. They also examined the performance of eigenanalysis in diverse demographic scenarios. In the following, we discuss the problem of sample selection bias and some related work about the effect of biased sampling on accuracy of population stratification.

### Sample selection bias

A common assumption of statistical algorithms is that the available sample is representative of the underlying population. In reality, this assumption may not always be true. Sample selection bias is any systematic difference between the sample and the population. It affects the internal validity of an analysis by leading to inaccurate estimation of relationships between variables. It also can affect the external validity of an analysis because the results from a biased sample may not generalize to the population.

The problem of sample selection bias was first widely studied in econometrics, where it appeared as a bias among survey responders. Heckman (1979) provided a method of addressing this problem in linear regression models by estimating the probability of an individual being included in the sample. Sample selection bias also has been addressed in the statistics and machine learning literature by attempts to understand its effect on classifiers and how estimation and prediction can be made correctly in the presence of sampling bias (Vella 1998; Zadrozny 2004; Davidson and Zadrozny 2005; Cortes *et al.* 2008). Zadrozny (2004) discusses the properties of learning algorithms and the effect of sample selection bias on their accuracy. It also outlines a possible way of correcting for sample selection bias, provided we know the bias. Sample selection bias is also studied in ecology when one is trying to model species distributions using presence-only data (Phillips *et al.* 2009).

In population genetics, sample selection bias could be a serious problem because the estimates of ancestry obtained from stratification analyses often are used to make inferences about the genetic ancestry of the sampled individuals. The inferred individual ancestries also are used as input in correcting for stratification in association studies [for instance, in ADMIXMAP (Hoggart *et al.* 2003)]. Pritchard *et al.* (2000) suggest that detecting stratification is difficult unless a significant number of unmixed individuals from each ancestral (or pseudo-ancestral) population is present in the sample. This observation was verified by Tang *et al.* (2005) through experiments on a small number of simulated datasets. To our knowledge, there has not been a systematic study of the effect of sample selection bias on the accuracy of population structure recovery and individual ancestry recovery.

We propose to study the effect of sample selection bias on the accuracy of population stratification and individual ancestry recovery using a model-based approach (ADMIXTURE) and an eigenanalysis-based approach (EIGENSOFT). Since the analysis of McVean (2009) provides guidelines on the effect of sampling bias on stratification accuracy using eigenanalysis, we will focus our attention mainly on probabilistic models such as ADMIXTURE.

## A mathematical framework for sample selection bias

We will consider the problem of studying genotype data using a probabilistic model. Ordinarily, for probabilistic modeling, we would assume that we have genotypes (*g*) drawn independently from a distribution *D* (with domain G) over the feature space G. We assume that our points (*g*, *u*, *s*) are drawn independently from a distribution *D* over G × U × S, where *G* is the space of genotypes, *U* are some auxiliary features of the data that are not of direct interest for modeling and *S* is a binary space. The variable *s* controls the selection of points (1 means the point is selected and is observed in our sample, 0 means the point is not selected). Our observed sample contains only points that have *s* = 1. We will refer to this as the selected sample and refer to its distribution as *D*′.

We consider the setting where *s* is independent of *g* given *u*, that is *P*(*s*|*g*, *u*) = *P*(*s*|*u*). This setting, where the selection is controlled by features different from the genotype we want to model, arises frequently in real applications. In population genetics, whether an individual is included in a genotyping study often depends on factors such as geographical location.

### Sample selection bias correction

It is easy to see that if *g* is independent of *u* in the previous setting, then sample selection bias has no effect and the probability of *g* in the selected sample is the same as probability of *g* under *D* (asymptotically). If *g* and *u* are not independent, then we can write using Bayes rule:

$$P\left(g,u\right)=\frac{P\left(s=1\right)P\left(g,u|s=1\right)}{P\left(s=1|g,u\right)}$$ (1)

$$=\frac{P\left(s=1\right)P\left(g,u|s=1\right)}{P\left(s=1|u\right)}$$ (2)

which can be rewritten as

$$P_{D}\left(g,u\right)=\frac{P\left(s=1\right)P_{D^{\prime }}\left(g,u\right)}{P\left(s=1|u\right)}$$ (3)

where *D*′ represents the distribution of the selected sample. Since the term *P*(*s* = 1) is constant with respect to (*g*, *u*), we can say that

$$P_{D}\left(g,u\right)=\frac{c\times P_{D^{\prime }}\left(g,u\right)}{P\left(s=1|u\right)}$$ (4)

where *c* is a constant that need not be evaluated for tasks such as learning model parameters. Therefore, to model *P_D_*(*g*, *u*) accurately to a multiplicative constant, we can follow the procedure below:

1. Compute *P_D_*_′_(*g*, *u*) using a model learned on the selected sample.
2. Apply a correction using the term *P*(*s* = 1|*u*). This can be done in two ways:
  (a) If we know the selection procedure, we know *P*(*s* = 1|*u*) and can directly use it in the model.
  (b) If the selection procedure is not known, but we have access to large number of points for which we know (*u*, *s*), but not *g*, we can estimate *P*(*s* = 1|*u*). In the population genetics example, this would correspond to knowing the language or geographical region of an individual and whether or not they could have been included in the study (since genotyping individuals to find *g* for a large number of individuals would be expensive).

However, this analysis, which can accurately correct for sample selection bias in the described setting, requires a model of both *g* and *u*. In most applications, we are interested in only modeling *g* and not *u*. For instance, although there is interest in modeling the distribution of genotypes, distributions of language or geography are not of direct interest in genetics. Therefore, we consider a similar analysis in the case where we only model *P*(*g*) and attempt to derive a correction for sample selection bias.

### Approximate correction

We consider the case when we only want to model *P*(*g*). Proceeding in a similar way as before, we can write

$$P\left(g\right)=\frac{P\left(s=1\right)P\left(g|s=1\right)}{P\left(s=1|g\right)}$$ (5)

which can be restated as

$$P_{D}\left(g\right)=\frac{c\times P_{D^{\prime }}\left(g\right)}{P\left(s=1|g\right)}$$ (6)

A correction for sample selection bias could therefore be found if we could estimate *P*(*s* = 1|*g*). However, *g* is typically high-dimensional—in genetics applications, *g* may have dimensions from 1000 to 1,000,000. *P*(*s* = 1|*g*) is therefore hard to estimate from the small selected sample. We propose that since *g* and *u* are dependent and *u* typically has much lower dimensionality than *g*, we can approximate *P*(*s* = 1|*g*) by *P*(*s* = 1|*u*). We can therefore write the correction for sample selection bias as

$$P_{D}\left(g\right)\approx \frac{c\times P_{D^{\prime }}\left(g\right)}{P\left(s=1|u\right)}$$ (7)

with the quality of the approximation varying as a function of the dependence between *g* and *u*. In practice, we find that the approximate correction method is adequate for most applications, since probabilistic models are often robust to some differences between the true distribution of the data and the distribution of the selected sample.

It is important to note that even if the selection is determined in reality by the *u* variables only, the correction proposed in Equation 7 is only an approximate correction. The exact correction would require computing the term *P*(*s* = 1|*g*) which can be written as $\sum_{u}P\left(s=1|u\right)P\left(u|g\right).$ The second term is a distribution conditioned on *g* and is hard to specify due to the high dimensionality of *g*.

#### Implementing correction in learning:

Although applying the proposed correction for accurate probability modeling only requires an extra multiplication step, implementing the correction in learning models consistent with the true distribution is more complex. This problem is well-studied as cost-sensitive learning. A discussion of the ways in which the correction can be applied to classifiers can be found in Zadrozny *et al.* (2003). In this work, we will use sampling with replacement to implement the correction. To perform sampling with replacement, we sample points in the selected sample (with replacement) with probability proportional to their correction factor (1/*p*(*s* = 1|*u*)). If the selected sample contains *N* points, the probability of inclusion for the *i^th^* point is given as $\frac{1/p\left(s=1|u_{i}\right)}{\sum_{j=1}^{N}1/p\left(s=1|u_{j}\right)}$. Since we sample with replacement, our corrected sample can include non-unique points from the selected sample.

## Methods

We will demonstrate the effects of sample selection bias on the accuracy of ancestry recovery by using experiments on simulated and real data. We will also show how the approximate correction for sample selection bias is effective in practice.

### Simulation experiments

**Figure 1:**
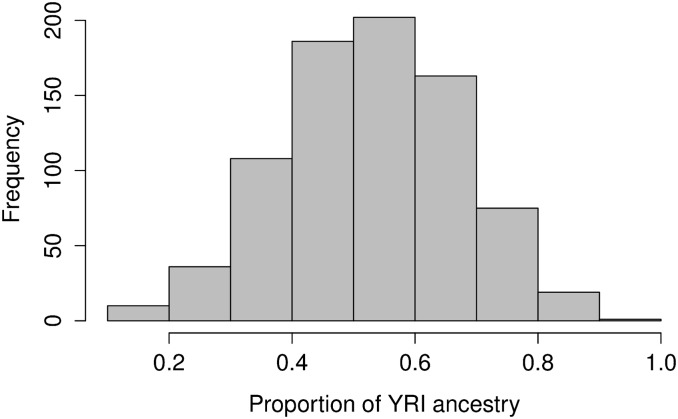
Proportion of YRI ancestry among individuals in the simulated population, six generations after admixture.

To examine the recovery of individual ancestry, we simulated data from a two-population admixture scenario using populations from HapMap Phase III as the ancestral populations. We used 50 unrelated individuals each from the CEU (Utah residents with ancestry from northern and western Europe) and YRI (Yoruba in Ibadan, Nigeria) populations as the two ancestral populations. Using a forward simulator (see File S1), we simulated a 50-50 admixture in a single generation followed by six generations of random mating to create a simulated population of 800 diploid individuals. For our experiments, we used a 102-Mb region from chromosome 1 containing 52,040 single-nucleotide polymorphisms (SNPs). The recombination rate was set to be 10^−8^ per site per generation and the mutation rate was set to be 10^−8^ per site per generation. Figure 1 shows the proportion of YRI ancestry among the individuals in the simulated population. From the figure, we can see that most individuals are admixed, and therefore we refer to this simulated population as admixed. For our ancestry inference experiments, we also included the remaining CEU-unrelated individuals (38 in total) and the remaining YRI-unrelated individuals (50 in total) in the analysis as proxies for the ancestral populations. In our experiments, we will refer to the CEU and YRI individuals as unmixed individuals.

We use the proportion of YRI ancestry, denoted as *θ_i_* to represent the ancestry of the *i^th^* individual. The population used for our experiments therefore contained the following three groups:

1. 100 CEU individuals obtained by duplicating (and in some cases triplicating) 38 unrelated CEU individuals, *θ_i_* = 0
2. 100 YRI individuals obtained by duplicating 50 unrelated YRI individuals, *θ_i_* = 1
3. 800 admixed individuals, with *θ_i_* ∈ (0, 1)

To study the effects of sampling bias, we sample individuals from the dataset with replacement to generate a dataset of size x + y + z, where x is the number of CEU individuals, z is the number of YRI individuals, and y is the number of admixed individuals. By varying x, y, and z, we can generate smaller datasets with different kinds of bias and deviations from the original dataset. We choose x and z from {10,30,50,100} and y from {10,30,100,200,400,700}. Thus, the smallest possible dataset is {10,10,10} (30 individuals), and the largest possible dataset is {100,700,100} (900 individuals). We will use *S_xyz_* to refer to the dataset {*x*, *y*, *z*}.

In this case, *g* comprises the 52,040-dimensional genotypes of the individuals and *u* comprises the group memberships of each individual according to their ancestry (*u* ∈ {1, 2, 3} with the three groups as defined earlier). In general, the probability distribution of *u* may not be known. Therefore, as a first guess, we will assume that *u* has a uniform distribution, *i.e.*, $P\left(u\right)=\frac{1}{3}$ for any value of *u*. This assumption means that an individual (regardless of inclusion in the study sample) is equally likely to be from the CEU, YRI, or the admixed population. By design, *s* depends only on *u*, and we can write the probability distribution P(*s* = 1|*u*) as:

$$P\left(s=1|u=1\right)=\frac{P\left(u=1|s=1\right)P\left(s=1\right)}{P\left(u=1\right)}=\frac{x}{x+y+z}\frac{P\left(s=1\right)}{1/3}$$ (8)

$$P\left(s=1|u=2\right)=\frac{P\left(u=2|s=1\right)P\left(s=1\right)}{P\left(u=2\right)}=\frac{z}{x+y+z}\frac{P\left(s=1\right)}{1/3}$$ (9)

$$P\left(s=1|u=3\right)=\frac{P\left(u=3|s=1\right)P\left(s=1\right)}{P\left(u=3\right)}=\frac{y}{x+y+z}\frac{P\left(s=1\right)}{1/3}$$ (10)

which we can simplify to write

P(s=1|u=1)=x×C (11)

P(s=1|u=2)=z×C (12)

P(s=1|u=3)=y×C (13)

where *C* is a constant given by $\frac{3P\left(s=1\right)}{x+y+z}$.

### Evaluation measure

A fair evaluation of the results for both ADMIXTURE and EIGENSOFT is difficult to achieve because the individual ancestries produced by ADMIXTURE and EIGENSOFT are different in nature. With *K* ancestral populations, an individual ancestry vector produced by ADMIXTURE has the form {*q*_1_, …, *q_k_*} such that $\sum_{k=1}^{K}q_{k}=1$. Thus, it has only *K* − 1 independent components. An equivalent representation of ancestry can be produced by projecting an individual on the first *K* − 1 eigenvectors produced by EIGENSOFT. The ancestry vectors that we store as the true ancestry when generating the simulation data have the same form as those produced by ADMIXTURE.

We use squared correlation between the true ancestry and inferred ancestry as the measure for evaluating the accuracy of ancestry inference. For the dataset *S_xyz_*, let Θ = {*θ*_1_, …, *θ_x_*_+_*_y_*_+_*_z_*} denote the true proportion of YRI ancestry of the individuals. Let *Q*_1_ = {*q*_1,1_,…,*q*_1,_*_x_*_+_*_y_*_+_*_z_*} denote the ancestry proportions in cluster 1 inferred by ADMIXTURE for dataset *S_xyz_* with *K* = 2, where the second subscript indexes the individuals in the dataset (and therefore we have that *Q*_2_ = {*q*_2,1_,…,*q*_2,_*_x_*_+_*_y_*_+_*_z_*} = {1 − *q*_1,1_, …, 1 − *q*_1,_*_x_*_+_*_y_*_+_*_z_*}. Then the accuracy metric for ADMIXTURE is *correlation*(Θ, *Q*_1_)^2^ (which is equal to *correlation*(Θ, *Q*_2_)^2^). The corresponding accuracy measure can be constructed for EIGENSOFT by replacing *Q*_1_ by the projections of the individual genotypes on the first eigenvector of the *S_xyz_* genotype matrix. For robustness, we report the mean and standard error of the squared correlation over 30 datasets for each parameter setting.

## Results

### Sample selection bias in a simulated CEU-YRI admixture

**Figure 2:**
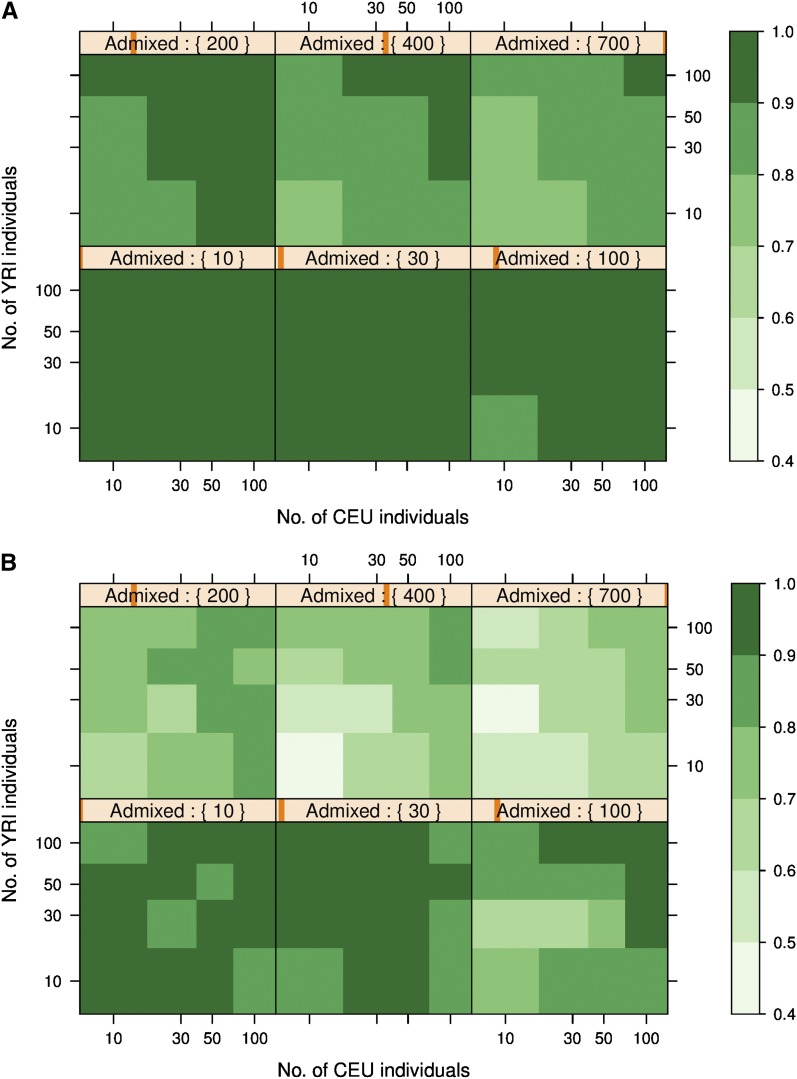
Squared correlation between the true individual ancestry and the individual ancestry inferred using (A) ADMIXTURE with K = 2 and (B) EIGENSOFT with the top eigenvalue. The different levelplots are drawn for different number of admixed individuals in the dataset. The X and Y axes of the plots are logarithmic in scale.

We examined the effect of biased sampling of individuals by constructing subsets of the whole dataset from the CEU-YRI admixture described earlier and measuring the squared correlation between the true and inferred ancestry (File S2). Figure 2A shows the results of this analysis with the ADMIXTURE software with six subplots. Within each subplot, the number of admixed individuals in the sample remains constant and the number of unmixed individuals from the two ancestral populations (CEU and YRI) is varied. Figure 2B shows the results of an identical analysis of the same datasets with the EIGENSOFT software.

Overall, ADMIXTURE recovered individual ancestry better than EIGENSOFT. Previous work has shown that the accuracy of individual ancestry recovery is a function of the *F_ST_* differentiation between the ancestral populations. The results we obtain may vary for datasets with different *F_ST_* values between the ancestral populations and quantifying this effect will require more study.

Figure 2B shows that the EIGENSOFT results exhibit considerable irregular variation within subplots. This is likely to be a result of our sampling scheme for creating biased datasets, which allows individuals to be present in a dataset more than once. This means that the resulting genotype matrix can have lower rank than expected, resulting in lower accuracy of ancestry inference using EIGENSOFT. This, however, does not cause any difficulties in the ADMIXTURE analysis, as seen by the regular patterns in Figure 2A and would be expected based on the likelihood model underlying ADMIXTURE. We will therefore only use the results in Figure 2B to draw broad conclusions about the behavior of EIGENSOFT and not make specific inferences or recommendations.

In the scenarios in which we have few unmixed individuals from both ancestral populations, Figure 2, A and B show that the accuracy of individual ancestry recovery drops noticeably. This effect is evident in the subplots with 200, 400, and 700 admixed individuals. In all subplots in Figure 2A, the results show high accuracy when we have a large number of unmixed individuals from at least one ancestral population. Pritchard *et al.* (2000) and Tang *et al.* (2005) have previously noted that a significant number of unmixed individuals from each ancestral population is required for accurate recovery of stratification. An initial examination of the results suggests that may be sufficient to have a large number (around 50−100) of unmixed individuals from just one of the two ancestral populations to be able to correctly resolve stratification.

We note that this guideline, which is relevant when the number of admixed individuals is large, does not apply if the dataset contains few admixed individuals and few unmixed individuals. When there are few admixed individuals, both methods perform well (relative to their average performance) even with as few as 10 unmixed individuals in the dataset. Mantel tests reveal that the high correlations obtained with few admixed individuals are statistically significant (*P* < 10^−3^) in all cases.

**Figure 3:**
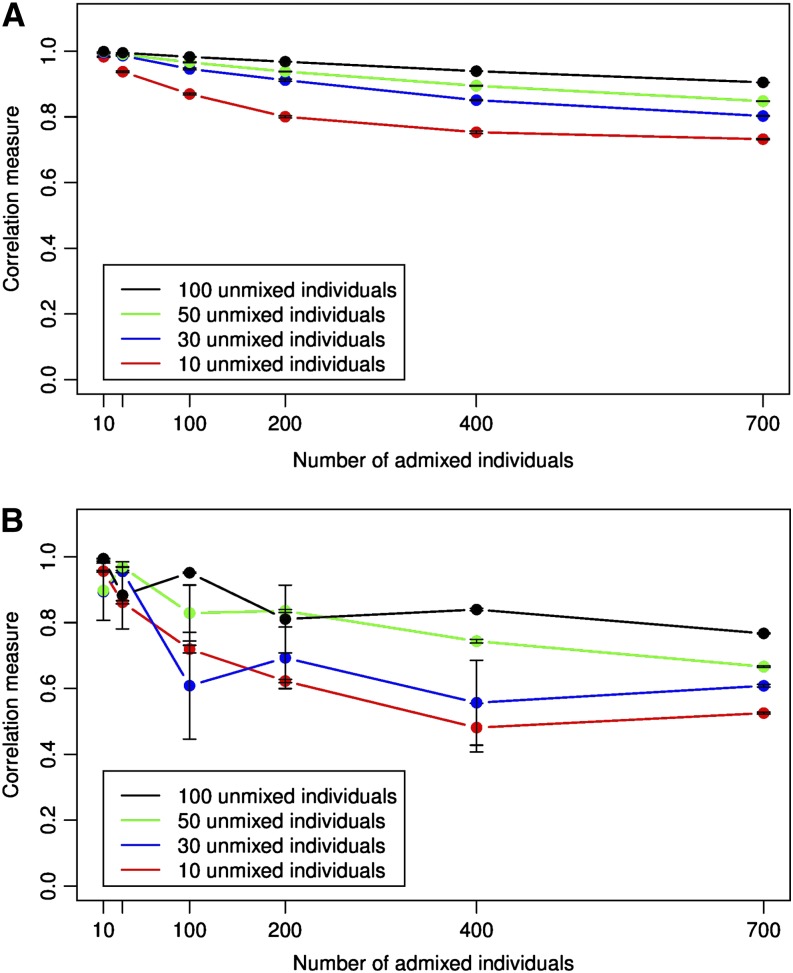
Effect of adding more admixed individuals to the dataset on the correlation measure of accuracy when using (A) ADMIXTURE with K = 2 and (B) EIGENSOFT with the top eigenvalue. The error bars indicate standard error.

To examine the effect of the number of unmixed individuals on the ancestry recovery in more detail, we looked at the subset of the generated subsamples which had the same number of unmixed individuals from both ancestral populations, *i.e.*, subsets of the form *S_xyz_* where the number of unmixed individuals x∈{10,30,50,100} and the number of admixed individuals y∈{10,30,100,200,400,700}. For each value of *x* (the number of unmixed individuals from each ancestral population present in the dataset), we observed the effect of varying *y* (the number of admixed individuals present in the dataset) on the ancestry recovery. Figure 3A shows the results for ADMIXTURE, and Figure 3B shows the results for EIGENSOFT. When the number of unmixed individuals is large, the methods recover ancestry well, and the number of admixed individuals has no effect on accuracy. However, when the number of unmixed individuals in the sample is small, adding more admixed individuals to the sample reduces the accuracy of the ancestry recovery for both ADMIXTURE and EIGENSOFT. In Figure 3, A and B, we see a threshold effect due to the number of unmixed individuals when the number of unmixed individuals changes from 30 to 100.

The high accuracy of ancestry recovery when there are few admixed and few unmixed individuals suggests that earlier hypotheses about the requirement of a large number of unmixed individuals for accurate ancestry recovery may be an incomplete explanation. The results in Figure 2A and Figure 3A, along with the likelihood model underlying ADMIXTURE suggest that the effect on accuracy may depend on the ratio of the number of admixed individuals to unmixed individuals from each population in the sample. For notational convenience, we will refer to this ratio as *τ_sample_* = *y*/*x*.

**Figure 4:**
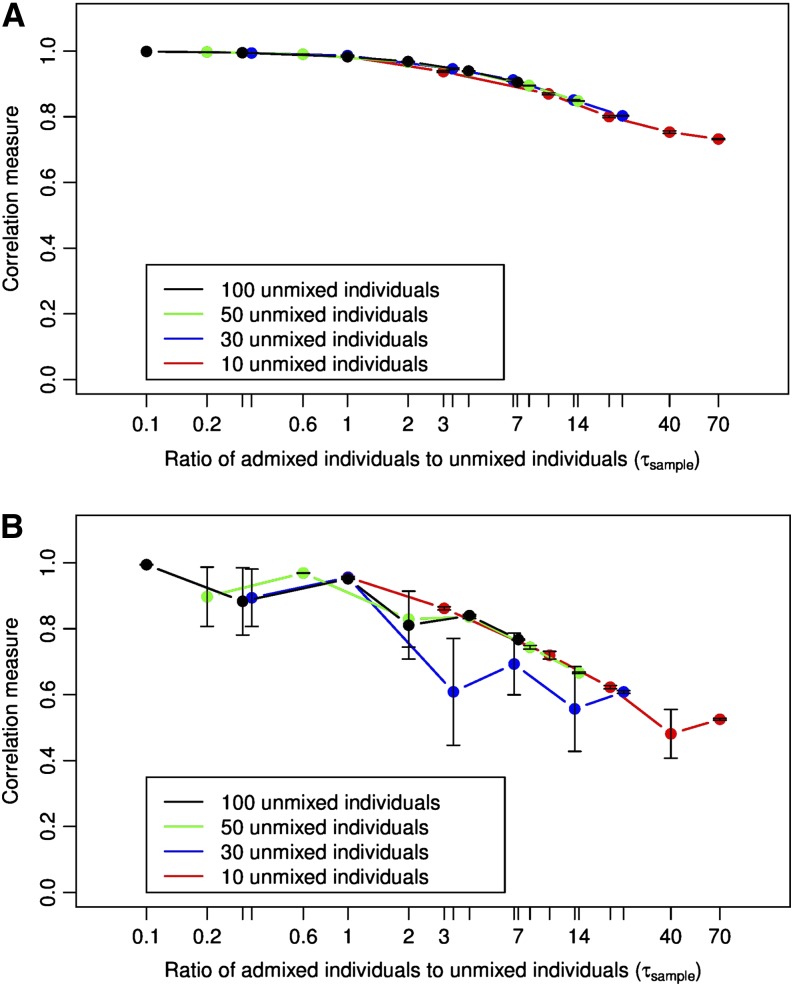
Effect of the ratio of number admixed individuals to unmixed individuals in the dataset (*τ_sample_*) on the correlation measure of accuracy using (A) ADMIXTURE with K = 2 and (B) EIGENSOFT with the top eigenvalue. The error bars indicate standard error. The X-axis is logarithmic in scale.

To examine this hypothesis, we replot the data used for Figure 3A by examining the correlation measure as a function of the ratio of admixed individuals to unmixed individuals in the sample. Figure 4, A and B show the results for this visualization for ADMIXTURE and EIGENSOFT, respectively. From the figure, we can see that the effect of sample selection bias can be understood using *τ_sample_*. The accuracy of ancestry recovery is high while the value of *τ_sample_* is less than 5 (approximately) and drops as this ratio increases. This behavior is independent of the exact number of unmixed individuals in the dataset and can be observed for both ADMIXTURE and EIGENSOFT.

In our experiments, we observe that individual ancestry can be recovered perfectly as long as *τ_sample_* < 5. Decreasing the number of admixed individuals has no adverse effect on the accuracy of ancestry recovery. The effects of sample selection bias on the accuracy of ancestry recovery in a two-population admixture scenario using ADMIXTURE can thus be explained in two scenarios: (i) when *τ_sample_* < 5, sample selection bias has no effect on the accuracy of individual ancestry recovery and (ii) when *τ_sample_* > 5, the accuracy of individual ancestry measured using the correlation measure decreases with *τ_sample_*.

#### Correction of sample selection bias in simulated data:

We examined three methods of correcting sample selection bias in the simulated data. The methods and their results are described herein.

##### Supervision:

ADMIXTURE can be run in supervised mode, where individuals of known ancestry can be specified in advance to belong to exactly one of *K* populations being inferred. This supervision is partial since only nonadmixed individuals can be part of the supervised input provided to the algorithm. In our experiments, we include supervision by assigning the individuals of known YRI and CEU ancestry to the two ancestral populations. We find that supervision produces an improvement that is statistically significant but practically insignificant (< 0.0001% improvement in squared correlation, *P* < 2.2 × 10^−16^ in a one-sided paired *t*-test).

##### Including more SNPs:

Our analysis of the CEU-YRI admixture used 52,040 SNPs in a 102-Mb region. We found that including more SNPs in the ADMIXTURE analysis reduced the effects of sample selection bias for identical sample sizes and samples. When 116,415 SNPs are included in the ADMIXTURE analysis, we observe no effects of sample selection bias on the accuracy of ancestry inference. Although we do not thin SNPs in our analysis, thinning the original set of 52,040 SNPs by an *r*^2^ threshold of 0.1 produces a set of 35,500 SNPs. Reanalyzing the data with this subset of SNPs produces a small loss in accuracy (<0.5% in squared correlation, *P* < 2.2 × 10^−16^ in a one-sided paired *t*-test) when compared with the original set of 52,040 SNPs. Thus, while adding independent SNPs improves accuracy of ancestry inference, adding or removing linked SNPs has little effect on accuracy.

##### Resampling correction:

We implemented the resampling correction in the section *Approximate correction* by using Equations 11−13. Since we do not have an informative prior expectation of the true numbers of admixed individuals or the unmixed individuals, we used the uninformative initial estimate of all groups of individuals having equal population sizes. We found that in more than 99% of the corrected datasets, the squared correlation between true and inferred ancestry was larger than 0.95 (mean = 0.98, standard deviation = 0.01). Thus the resampling correction is effective for correcting sample selection bias.

### Impact of effective population size on sample selection bias

In the previous simulation, we used a correction based on an assumption of similar population sizes for the different groups. In general, the census (observed) population size and the effective population size of a population need not be equal due to demographic events that the population may have undergone. We attempt to demonstrate that the effect of biased sampling is dependent on the effective population size and not only on the census population size.

**Figure 5:**
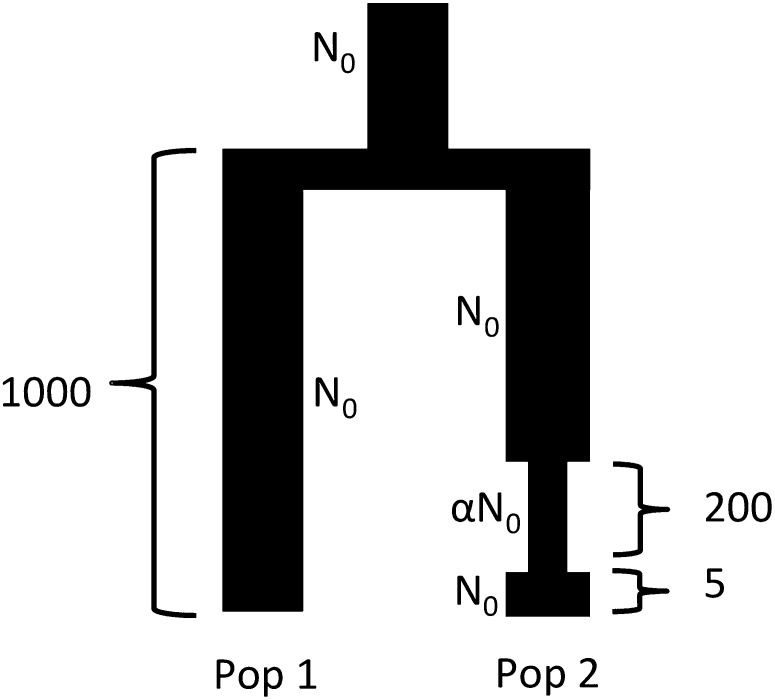
A two-population demographic scenario: Populations 1 and 2 (of size *N*_0_ = 10^4^) are formed from a single population of the same size 1000 generations before present time. Population 2 then undergoes a bottleneck for 200 generations followed by a instantaneous recovery to its original population size.

We simulated two populations according to the demographic scenario shown in Figure 5 using the coalescent simulator *ms* (Hudson 2002). The founding population and the populations resulting from the split 1000 generations before present were set to have identical sizes (*N*_0_ = 10,000). Population 2 then undergoes an instantaneous contraction 795 generations after the split, with its population size being reduced to *αN*_0_ The contraction factor *α* was varied across datasets, taking values of 0.99 (nearly no contraction), 0.5 (moderate contraction), or 0.1 (strong contraction). Two-hundred generations after the contraction, population 2 instantaneously grows back in size to *N*_0_. The simulation was continued for five generations after the recovery, after which 50 diploid individuals were sampled from each population. Each simulation used a mutation rate and recombination rate of 10^−8^ per bp per generation. In each simulation, 200 independent sites of 100 kb were generated, producing between 47,585 and 58,024 SNPs per simulation. For each value of *α* (from {0.1,0.5,0.99}), 30 independent datasets were generated to evaluate the robustness of results (File S3).

The two populations from a single simulation were pooled together to form a 50:50 admixture in a single generation to create 50 admixed individuals. This was followed by five generations of random mating in the resulting admixed population, using the simulator described in the *Introduction* section. We then generated a sample for study by including the 50 individuals from population 1, 50 admixed individuals, and a variable number of individuals (*x*) from population 2 (*x* ∈ {5, 10, 20, 30, 40, 50}). ADMIXTURE was used with *K* = 2 to infer the ancestry proportions for each individual and the squared correlation coefficient (as described in the section *Evaluation measure*) for the dataset was used a measure of accuracy.

**Figure 6:**
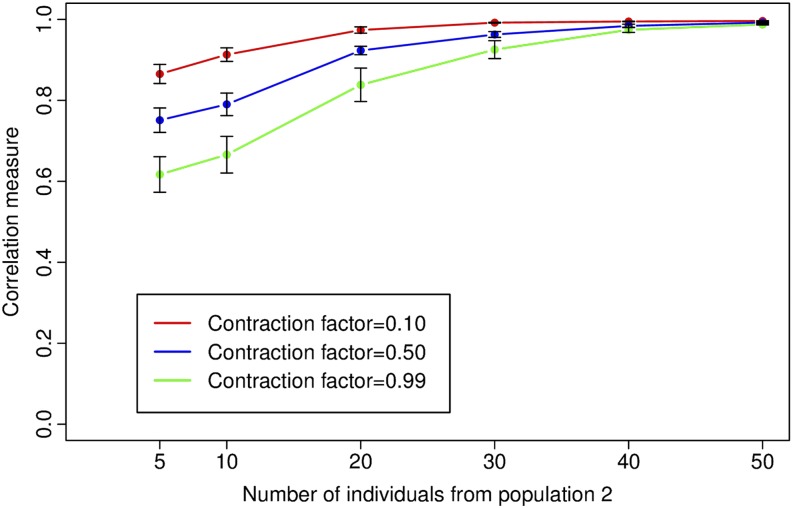
Impact of effective population size on the correlation measure of accuracy of ancestry inference using ADMIXTURE with K = 2. Each curve is drawn for a single value of the contraction factor *α* and shows the mean and standard error of the correlation measure over 30 datasets.

Figure 6 shows the results of the ancestry inference, with one curve for each value of the contraction factor *α*. As expected from the previous experiments, as the number of individuals from population 2 in the dataset increases, the accuracy of ancestry inference increases, which is demonstrated by each curve. We also observe that the curves for the three different contraction factors have different accuracies before they converge to a accuracy of almost 1.

By our experimental design, the two ancestral populations have identical census population sizes for each simulation and the census size for population 2 is identical across simulations. However, due to the bottleneck, the effective population size of population 2 is reduced, with the amount of reduction dependent to the strength of the contraction (the calculations for reduction in effective population size for population 2 can be found in File S1). Figure 6 thus demonstrates that effective population size, and not just census population size, has an impact on how ancestry inference is affected by sampling bias. From the figure, we can see that for the strongest contraction (*α* = 0.1, producing the maximum reduction in the effective population size for population 2 to 0.36*N*_0_), the accuracy of ancestry inference is high even with only 5 individuals from population 2. Since effective population size is a measure of the genetic diversity of a population, this suggests that the reduced genetic diversity produced by the strong contraction can be adequately captured even with a small number of individuals. For the same number of individuals from population 2, across the curves, we see that the accuracy is smallest for the weakest contraction (*α* = 0.99) and largest for the strongest contraction (*α* = 0.1). Thus, even when census population sizes are identical, variation in effective population size affects the impact of sampling bias accuracy of ancestry inference. This suggests that sampling bias can be more accurately captured by defining it in terms of effective population size rather than census population size.

#### Correction of bias:

**Figure 7:**
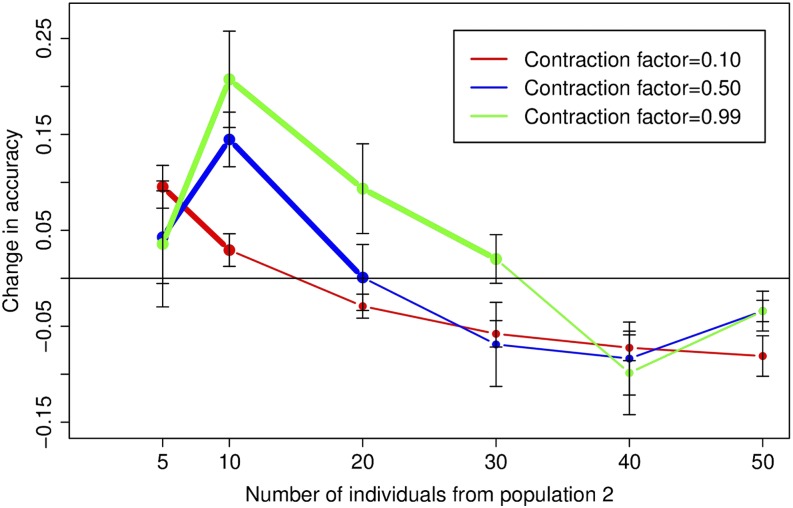
Change in correlation measure from Figure 6 after the resampling correction, taking into account the impact of the contraction on effective population size. Each curve is drawn for a single value of the contraction factor *α* and shows the mean and standard error of the change in correlation measure over 30 datasets. The regions of the curves in bold show regions where accuracy in the uncorrected results (from Figure 6) is less than 0.95.

Using the estimate for the effective population size of population 2 from File S1, we can implement a resampling correction as before. We assume that the effective population size for the admixed population is identical to that for population 1, *i.e.*, *N*_0_ = 10^4^. Figure 7 shows the change in accuracy after application of the resampling correction (relative to the accuracies in Figure 6).

From the figure, we can see that while the accuracy for the original dataset is less than 0.95, the resampling correction produces an improvement in the results, denoted by the portions of the curves in bold. However, beyond those points, as the datasets acquire an excess of the unmixed individuals relative to the admixed individuals, we see that the uncorrected accuracy is high (larger than 0.95) and the resampling correction decreases the accuracy of ancestry inference. Thus a resampling correction is unnecessary when there is an excess of unmixed individuals relative to their expected number from effective population size calculations.

### Sample selection bias in cattle genotype data

To demonstrate the effects of sample selection bias on a real genetic dataset, we used the data from (McTavish *et al.*, 2013a,b), who genotyped 1461 individuals belonging to cattle breeds across the world at 47,506 SNPs. We focus on admixture between African and European cattle breeds as seen in their New World descendants. For their experiments with STRUCTURE, McTavish *et al.* (2013b) used a subset of 1814 SNPs that was common to all their genotyping chips. Our data therefore consists of genotype data at 1814 SNPs from 40 New World individuals (Texas Longhorns), 100 European individuals (Limousin from Southern Europe), and 100 African individuals (N’Dama and N’DamaXBoran).

We created multiple subsets from the original data by including all the 40 admixed New World individuals and a variable number (*x*) of unmixed individuals from the European and African populations (with the same number from each population). The number of unmixed individuals chosen was increased gradually from 10 to 100 (*x* ∈ {10, 20, 30, 40, 50, 60, 80, 100}). For each value of *x*, we generated 30 datasets for statistical robustness. ADMIXTURE was used with *K* = 2 to infer ancestry proportions for all individuals. The squared correlation coefficient for the ancestry proportions of the admixed individuals was used as a measure of accuracy, with the ancestry proportions inferred on the full data used as a truth set.

**Figure 8:**
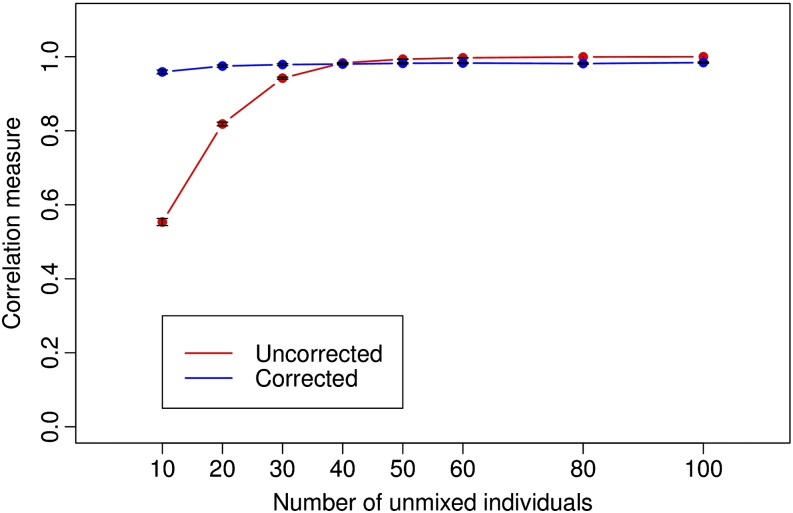
Variation in accuracy of ancestry inference with changing number of unmixed individuals. The red curve shows accuracy without correction and the blue curve shows the accuracy when the correction is applied.

Figure 8 shows the results of the ancestry inference as a function of the number of unmixed individuals (*x*). We see that as the number of unmixed individuals increases, the accuracy of ancestry inference improves. When the number of unmixed individuals is less than 40, the squared correlation measure is less than 0.9.

#### Correction of bias:

For the cattle data, effective population sizes for the three populations are unknown. We assume that all three populations have equal effective population size. The correction is implemented using resampling as before. The blue curve in Figure 8 shows the effect of the correction on accuracy. From the figure, we can see that when the number of admixed individuals is larger than the number of unmixed individuals (*τ_sample_* ≥ 1 as defined previously), the correction improves accuracy but causes a small drop in accuracy when there are many unmixed individuals.

## Discussion

Biased sampling of individuals is a result of practical constraints on the study design of genotyping/sequencing projects. In this aspect, sample selection bias is similar to bias due to SNP ascertainment. It can also occur as a result of combining multiple genomic datasets from different sources. For instance, the overlap of three genotyping chips required McTavish *et al.* (2013b) to use only 1814 SNPs for their STRUCTURE analysis.

In most stratification analyses, the recovered ancestry proportions are used to make inferences about the ancestry of the sample and the genetic relationships of similarity/differences between the study populations. Ancestry proportion estimates are also used in association studies to account for effects of stratification. Therefore, it is essential to have accurate recovery of ancestry. Our experiments suggest that sample selection bias can be a problem in accurate population stratification and recovering individual ancestry. Our simulations show that unlike observations from previous studies, the accuracy of individual ancestry recovery is not dependent only on the number of unmixed individuals present in the sample. We observe a threshold effect where the accuracy of ancestry inference is affected by sample selection bias depending on the ratio of admixed individuals to unmixed individuals in the sample. Our simulations suggest that the accuracy of ancestry inference is affected when this ratio, *τ_sample_*, is less than 5. For real genotype data from cattle, we observed that a ratio of *τ_sample_* larger than 1 was sufficient to affect ancestry estimates.

Our experiments do not directly demonstrate an effect of *F_ST_* on sample selection bias. However, using the 1814 SNPs for the genotype data, we were able to observe effects of sample selection bias on African-European admixture in cattle (*F_ST_* ≈ 0.15) but unable to observe any effect of sample selection bias on European-Indian admixture (*F_ST_* ≈ 0.28, results not shown here). This suggests that *F_ST_* has an effect on sample selection bias, with well-differentiated populations being easy to separate even with biased sampling. The simulations and real data analysis had relatively large *F_ST_* between the two ancestral populations (≈ 0.1 for the simulations, ≈ 0.15 for the cattle genotype data) but vastly different number of SNPs (52,040 for the simulation but only 1814 for the cattle genotype data). The degradation in performance of ancestry inference in the two cases was considerably different, suggesting that the effect of sample selection bias also depends on the number of loci available. This was also validated by the observation that doubling the number of SNPs in simulation eliminated the effects of sample selection bias completely.

We also showed through simulations that sample selection bias depends on the effective population size rather than simply the census population size of the populations studied. This suggests that sampling for genotyping/sequencing projects can be improved (in terms of diversity of sampling and cost) by taking into account independent knowledge of the demographic history of the populations being sampled.

Although our analyses use two specific methods (ADMIXTURE and EIGENSOFT), the effects we observe are a feature of the assumptions underlying both methods rather than the specific implementations. ADMIXTURE is a representative of the likelihood-based models that assume (a) admixture between ancestral populations and (b) that modern individual genomes are mixture of contributions from different ancestral populations. This is the model underlying STRUCTURE and frappe and to a large extent the extensions mentioned earlier. Likelihood-based methods are susceptible to effects of sample selection bias since each individual is given equal weight in the sample a priori. This is a result of the fundamental assumption of learning methods that the sample observed is representative of the underlying population distribution. Eigenanalysis, which also weighs each individual equally apriori, also suffers from a similar problem as the likelihood-based method. The sensitivity of eigenanalysis to sample size variation and outliers is well-known and is also reported by McVean (2009) and Novembre and Stephens (2008).

ADMIXTURE and EIGENSOFT are unsupervised methods for performing ancestry inference, *i.e.*, they do not leverage information about individuals of known ancestry to estimate their respective model parameters. ADMIXTURE supports a mode (which is referred to as a supervised mode in the documentation) in which some individuals can be “labeled”, *i.e.*, assigned to have ancestry from only a single cluster. However, all the individuals, labeled or otherwise, are used to learn the allele frequency parameters of the ADMIXTURE model. In machine learning literature, this mode of learning is commonly called “semi-supervised” learning, wherein a combination of labeled and unlabeled data is used to learn model parameters. In simulations, we observed that this mode of ancestry inference is also affected by sample selection bias.

A different class of methods can estimate local (subcontinental) ancestry from dense genotype data (Sankararaman *et al.* 2008; Price *et al.* 2009; Wall *et al.* 2011; Baran *et al.* 2012). A common theme of all of these methods is the use of external sources of genotype data as “reference panels” (referred to as “training data” in machine learning literature), which are used to estimate ancestral allele frequencies or ancestral haplotypes. The estimated parameters (frequencies or haplotypes) are then used to infer the ancestry of the individuals in the study sample based on their genotypes (referred to as “test data” in machine learning literature). Therefore, these methods can be said to be performing supervised learning. Supervised learning using external sources of data for estimating model parameters is robust to biased sampling in the study sample but is sensitive to the reference panels used for training (Zadrozny *et al.* 2003).

We proposed a resampling correction for sample selection bias using a mathematical framework modeling the sampling process. The proposed correction requires knowledge of some auxiliary information about the selection criteria that is correlated with the genotypes (bias is present only if selection is dependent on the genotypes, directly or indirectly). For genetic datasets, geography provides one such criterion that is easy to acquire during data collection. Using this information, we proposed a correction that is easy to implement. We showed using simulation experiments that such a correction is effective in practice and leads to more accurate results. In terms of computational effort, ADMIXTURE has a linear dependence on the number of individuals. The resampling correction will therefore require time proportional to the number of individuals in the resampled set. However, a better implementation of the resampling correction for ADMIXTURE would be to be weigh the likelihood contribution from each individual in the original sample by its resampling correction factor (1/*p*(*s* = 1|*u*)). This would allow the correction to be implemented without any increase in computational complexity of the ADMIXTURE algorithm. The resampling correction implemented naively can cause problems for EIGENSOFT due to the possibility of colinearity resulting from duplication of individuals, as evident in Figure 2B. A weighted eigenanalysis would avoid this problem without affecting the efficiency of the analysis.

An alternative method for correcting sample selection bias is to add more markers to the analysis, as observed from the simulation. Adding more SNPs to the analysis is an appealing solution because it does not require any further assumptions that any statistical correction may require, with the exception of the assumption of no linkage between SNPs. However, this may not always be possible if the study dataset is constructed by intersecting multiple datasets containing the individuals of interest genotyped on different platforms.

Our simulation experiments used simple two-population admixture scenarios to examine the effects of sample selection bias on the accuracy of stratification. In one set of experiments, we used two populations (CEU and YRI) that were easily separable and examined the admixed population resulting after a single recent admixture event. In reality, the demographic processes underlying the evolution of populations are much more complicated. In such scenarios, it is reasonable to expect that the stratification problem may be harder to resolve and would suffer from the effects of sample selection bias more severely.

A limitation of our proposed correction is the requirement for external knowledge of the selection criteria for samples. In our experiments, we either knew the nature of the bias (by design in the simulation experiments) or assumed that it was known. In general, the accuracy of the correction method proposed will strongly depend on the relationship between the selection criteria and the genotypes. Corrections factors therefore may be dataset-specific. An alternative future direction for correcting sample selection bias would be to develop models of population structure that can also model the auxiliary factors such as geography or language that may determine the selection process responsible for the biased sampling. There may be occasions where the nature of the sampling bias is partially known without having sufficient information to allow a correction to be developed. For instance, analyses of the POPRES dataset (Nelson *et al.* 2008) often select individuals who are known to have all four grandparents of the same ancestry (Novembre *et al.* 2008). In this case, the criteria for selection can be stated explicitly but a mathematical formulation of the bias is hard to obtain. In such scenarios, we recommend subsampling/resampling datasets to examine the robustness of ancestry inference results to variation in sampling.

In summary, our experiments suggest that sample selection bias affects ancestry inference and depends on factors such as effective population size, genetic differentiation between populations, number of loci available, and the ratio of admixed samples to unmixed samples. For analyses of human genotype data, hundreds of thousands of SNPs are typically available. Therefore, we do not expect sample selection bias to be a problem for ancestry inference in human data except when the source populations for admixture are not well-differentiated. Local ancestry methods which can make use of external genotype data also provide a way of avoiding the effects of sample selection bias. We expect that sample selection bias will be of interest in ancestry analysis for model organisms, where availability of external genotype data are limited and most genotyping chips have relatively fewer loci. When the sampling process is relatively well-understood, a mathematical correction as we have proposed will help to reduce the effects of sample selection bias. In our experiments, a rough guideline of using approximately identical number of admixed and unmixed individuals reduces the effects of biased sampling. When the sampling process cannot be modeled accurately, examining the robustness of ancestry estimates to sampling may help avoid inaccurate ancestry estimation.

## 
